# PELI1 promotes radiotherapy sensitivity by inhibiting noncanonical NF‐κB in esophageal squamous cancer

**DOI:** 10.1002/1878-0261.13134

**Published:** 2021-11-14

**Authors:** Dongfang Dai, Hongping Zhou, Li Yin, Fei Ye, Xiao Yuan, Tao You, Xiaohui Zhao, Weiguo Long, Deqiang Wang, Xia He, Jifeng Feng, Deyu Chen

**Affiliations:** ^1^ Department of Radiotherapy, The Affiliated Cancer Hospital of Nanjing Medical University Jiangsu Cancer Hospital, Jiangsu Institute of Cancer Research Nanjing China; ^2^ Institute of Oncology Affiliated Hospital of Jiangsu University Zhenjiang China; ^3^ Department of Radiotherapy The Affiliated BenQ Hospital of Nanjing Medical University China

**Keywords:** NIK, noncanonical NF‐κB, PELI1, polyubiquitination, radiotherapy

## Abstract

The low sensitivity of radiotherapy is the main cause of tumor tolerance against ionizing radiation (IR). However, the molecular mechanisms by which radiosensitivity is controlled remain elusive. Here, we observed that high expression of pellino E3 ubiquitin protein ligase 1 (PELI1) was correlated with improved prognosis in human esophageal squamous cell carcinoma stage III patients that received adjuvant radiotherapy. Moreover, we found PELI1‐mediated IR‐induced tumor cell apoptosis *in vivo* and *in vitro*. Mechanistically, PELI1 mediated the lysine 48 (Lys48)–linked polyubiquitination and degradation of NF‐κB–inducing kinase (NIK; also known as MAP3K14), the master kinase of the noncanonical NF‐κB pathway, thereby inhibiting IR‐induced activation of the noncanonical NF‐κB signaling pathway during radiotherapy. As a consequence, PELI1 inhibited the noncanonical NF‐κB–induced expression of the anti‐apoptotic gene BCL2 like 1 (*Bclxl*; also known as *BCL2L1*), leading to an enhancement of the IR‐induced apoptosis signaling pathway and ultimately promoting IR‐induced apoptosis in tumor cells. Therefore, *Bclxl* or *NIK* knockdown abolished the apoptosis‐resistant effect in *PELI1*‐knockdown tumor cells after radiotherapy. These findings establish PELI1 as a critical tumor intrinsic regulator in controlling the sensitivity of tumor cells to radiotherapy through modulating IR‐induced noncanonical NF‐κB expression.

## Graphical abstract

During radiotherapy, ionizing radiation (IR) promotes NIK accumulation and then noncanonical NF‐κB activation in esophageal squamous cancer (ESCC) cells, which in turn mediates the expression of *Bclxl*, and thereby suppresses IR‐induced tumor cell apoptosis. Additionally, PELI1 mediates Lys48‐linked polyubiquitination and degradation of NIK and inhibits IR‐induced activation of noncanonical NF‐κB signaling. Our findings identify PELI1 as a crucial regulator of tumor radiosensitivity in patients with ESCC.

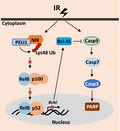

**Abbreviations**
4‐NQO: 4‐nitroquinoline 1‐oxide
^18^F‐FDG: 2‐Deoxy‐2‐^18^F‐fluoro‐D‐glucose
^18^F‐ML‐10: 2‐(5‐^18^F‐fluoropentyl)‐2‐methyl‐malonic acid
DAPI: 4′, 6‐diamidino‐2‐phenylindole
DFS: disease‐free survival
ESCC: esophageal squamous cell carcinoma
IR: ionizing radiation
NIK: NF‐κB‐inducing kinase
OS: overall survival
OSEM3D: three‐dimensional ordered‐subset expectation maximization
PELI1: pellino E3 ubiquitin protein ligase 1
PET/CT: positron emission tomography/X‐ray computed tomography
Quantitative RT‐PCR: quantitative Real‐Time PCR
ROS: reactive oxygen species
SUV: standard uptake value
WT: wild‐type

## 1 Introduction

Ionizing radiation (IR) is a conventional and powerful approach in the fight against cancer [1], and it still plays an important role in the clinical treatment of malignant tumors, such as esophageal squamous cell carcinoma (ESCC) [2], lung cancer [3], and breast cancer [4]. IR‐induced apoptosis of tumor cells is considered to be one of the basic principle methods of killing tumors during radiotherapy [1]. However, the modulation of the radiosensitivity of tumors has always been a difficult question in radiotherapy, and the molecular mechanism of radiotherapy‐induced apoptosis of tumor cells is also poorly understood. Noncanonical NF‐κB signaling has been implicated in regulating lymphoid organogenesis, B‐cell maturation, survival and activation, osteoclast differentiation, and diverse functions of other immune cells [5, 6, 7]. Accumulating evidence has demonstrated that aberrant activation of the noncanonical NF‐κB pathway is commonly observed in human malignancies [8, 9, 10, 11, 12, 13], including hematological and non‐hematological tumors, implying new regulatory roles for this pathway in cancer biology. However, the intrinsic function of noncanonical NF‐κB in ESCC to modulate radiosensitivity remains elusive.

The activation of noncanonical NF‐κB signaling depends on the accumulation of NF‐κB‐inducing kinase (NIK), which is a master kinase for the activation of this pathway and is tightly controlled by the ubiquitination system [5, 6, 7]. Published studies have shown that the E3 ubiquitin ligase PELI1 mediates the Lys48‐linked poly‐ubiquitination and degradation of NIK, and thus PELI1 deletion significantly promotes the accumulation of NIK, thereby inducing activation of non‐canonical NF‐κB pathway in B cells [14, 15]. In the immune system, PELI1 is critical for regulating the function of both innate and adaptive immune cells [14, 15, 16, 17, 18, 19]. Recent studies have demonstrated that PELI1 may contribute to the growth and chemoresistance of lung cancer cells through its Lys63‐mediated polyubiquitination [20, 21], suggesting a potential novel function of PELI1 in tumor biology. However, the biological functions of PELI1 in other types of tumors and in radiotherapy are largely unknown.

In the present study, we found that high PELI1 expression in ESCC was correlated with a favorable prognosis and beneficial result of adjuvant radiotherapy. Mechanistic studies have suggested that PELI1 functions as an essential mediator of IR‐induced tumor cell apoptosis by suppressing noncanonical NF‐κB signaling, leading to inhibition of the anti‐apoptotic protein Bcl‐XL and induction of the apoptotic signaling pathway during radiotherapy.

## 2 Materials and methods

### 2.1 Patient samples

A total of 331 ESCC cancer tissues and matched cancer‐adjacent normal tissue samples were collected at Affiliated Hospital of Jiangsu University from Jul 2010 to Dec 2013. None of the patients had received any type of treatment (radiation therapy, chemotherapy, or immunotherapy) before their operation. Patients with a pathological diagnosis of TNM stage III after R0 radical operation received adjuvant radiotherapy or observation, and their follow‐ups were completed in Dec 2018. The cancer and their adjacent tissues that used for histological analysis were collected before postoperative radiotherapy. The detailed information of patients can be found in Table 1. The patients' overall survival (OS) and disease‐free survival (DFS) durations were defined as the interval from initial surgery to death and from initial surgery to clinically or radiologically demonstrated recurrence or metastasis, respectively. The protocol of this study was approved by the Human Research Ethics Committee of Affiliated Hospital of Jiangsu University, and written informed consent was obtained from each patient. The study methodologies conformed to the standards set by the Declaration of Helsinki.

**Table 1:** Patient characteristics.

Patient characteristics	PELI1 expression^a^		*P* value
	Low (*n* = 144)	High (*n* = 187)	
Age (years)			
< 65	84 (58.3)	113 (60.4)	0.700
≥ 65	60 (41.7)	74 (39.6)	
Sex			
Female	43 (29.9)	60 (32.1)	0.665
Male	101 (70.1)	127 (67.9)	
Tumor location			
Upper thoracic	9 (6.3)	17 (9.1)	0.341
Middle and lower thoracic	135 (93.8)	170 (90.9)	
Lesion length (cm)^b^			
< 4	70 (48.6)	122 (65.2)	0.002
≥ 4	74 (51.4)	65 (34.8)	
Histology grade			
I/II	117 (81.3)	139 (74.3)	0.136
III	27 (18.8)	48 (25.7)	
TNM stage			
I/II	88 (61.1)	110 (58.8)	0.674
III	56 (38.9)	77 (41.2)	

^a^ The percentage of PELI1 positive cells was divided into five grades (percentage scores): < 10%, 0; 10–25%, 1; 26–50%, 2; 51–75%, 3; and > 75%, 4. The intensity of the PELI1 staining was divided into four grades (intensity scores): no staining, 0; light brown, 1; brown, 2; and dark brown, 3. The PELI1 positivity was determined using the following formula: overall scores = percentage score × intensity score. Overall scores of < 6 and ≥ 6 were defined as low and high expression, respectively.

^b^ Median length as a cutoff.

### 2.2 Mice

*Peli1*‐deficient mice (C57BL/6 background) were generated by Shanghai Model Organisms Center. *Peli1* ^+/−^ mice were bred to generate *Peli1* ^−/−^ (KO) and *Peli1* ^+/+^ (WT) mice, which were used in the experiments. For the induction of ESCC, 8‐week‐old female mice were fed drinking water containing 100 μg·mL^−1^ of 4‐NQO. The mice were allowed access to the drinking water at all times. After 16 weeks of 4‐NQO treatment, the mice were fed distilled water for another 12 weeks without 4‐NQO. For the tumorigenicity assay, *PELI1* overexpression or knockdown cells or control cells (5 × 10^6^; *n* = 4 mice/group) were injected subcutaneously into female athymic nude mice (6‐week‐old). Mouse tumor volume, weight, and survival data were recorded. All mice were maintained in a specific pathogen–free facility, and all animal procedures were approved by the Institutional Animal Care and Use Committee of Jiangsu University (UJS‐LAER‐2017071001).

### 2.3 Antibodies and reagents

The anti‐PELI1 (sc‐271065), anti‐NFκB p100/p52 (sc‐7386), anti‐RelB (sc‐48366), and anti‐Lamin B (sc‐6216) antibodies were purchased from Santa Cruz Biotechnology (Dallas, TX, USA), and anti‐NIK (4994), anti‐ACTIN (4970), anti‐Cleaved PARP (5625), anti‐Cleaved Caspase‐3 (9664), anti‐Cleaved Caspase‐7 (8438), anti‐Cleaved Caspase‐9 (52873), anti‐Bcl‐XL (2764), anti‐Bax (2772), anti‐Bcl‐2 (15071), anti‐Bak (12105), anti‐Mcl‐1(5453), anti‐A1/Bfl‐1 (D1A1C) (14093), anti‐Bcl‐w (31H4) (2724), anti‐Phospho‐NF‐κB p65 (Ser536) (3033), and anti‐Phospho‐Akt (Ser473) (4060) anti‐p53 (2527), anti‐Phospho‐p53 (Ser15) (9284), anti‐ATM (2873), anti‐Phospho‐ATM (Ser1981) (13050), anti‐NF‐κB1 p105 (4717), anti‐c‐Rel (4727), anti‐Phospho‐NF‐κB2 p100/p52 (Ser866/870) (4810), and anti‐Phospho‐RelB (Ser552) (D41B9) (5025) antibodies were purchased from Cell Signaling Technology (Danvers, MA, USA). The anti‐Lys48 ubiquitin (051307) antibody was purchased from Millipore (Burlington, MA, USA). MG132 (C2211) and 4‐NQO (N8141) were purchased from Sigma (St. Louis, MO, USA). Puromycin was purchased from Merck (54041, Kenilworth, NJ, USA). Cycloheximide (HY‐12320) was purchased from MCE. ^18^F‐FDG and ^18^F‐ML‐10 were synthesized by Jiangsu Huayi Technology Co., Ltd (Suzhou, China). The radiochemical purity was more than 95%.

### 2.4 Cell culture

Four human squamous cell carcinomas cell lines, ECA‐109, TE‐1, SCC‐9 and SiHa, were purchased from Shanghai Genechem Co., Ltd (Shanghai, China). and were tested for free of mycoplasma contamination. Cells were maintained in RPMI 1640 or MEM medium (Gibco, Carlsbad, CA, USA) containing 10% FBS (Gibco) and 1% penicillin‐streptomycin (Gibco) and cultured at 37 °C in 5% CO_2_. For the IR treatment, the cells received a single fraction of 10 Gy X‐rays irradiation by a linear accelerator (VARIAN 23EX, 6 MV X‐rays, a dose rate 400 cGy·min^−1^, Palo Alto, CA, USA). After irradiation, the cells were maintained in the CO_2_ incubator and analyzed at the indicated time points.

### 2.5 Gene knockdown and overexpression

For PELI1 knockdown, the HEK293T cells were transfected with the vector pLKO.1 or pLKO.1‐sh*PELI1,* pLKO.1‐sh*Bclxl,* or pLKO.1‐sh*NIK* vectors, along with the packaging vectors pMD2 and psPAX2. The lentiviral supernatants were collected 48 h after transfection and used for cancer cell infection and subsequent puromycin or GFP selection. For *PELI1* overexpression, the pRV‐GFP retrovirus encoding the PELI1 vector, C‐terminal truncated PELI1 (PELI1∆C) or empty vector were used to transduce the cancer cells. The infected cells were then enriched by flow cytometric sorting on the basis of GFP expression.

### 2.6 Clonogenic assay

The radiosensitivity of TE‐1 and ECA‐109 cells was assessed by cell colony‐formation assay. Cells were plated in six‐well plate at a density of 200, 400, 600, 1000, and 1500 cells/well and then exposed to 0, 2, 4, 6, and 8 Gy IR, respectively, the next day. The surviving cells colonies were fixed with 2% paraformaldehyde and stained with trypan blue and were counted. The survival fraction (SF) was calculated based on the formula SF = 1 − (1 − *e* ^−^ *^kD^*)*^n^* to generate dose‐survival curves.

### 2.7 Immunoblot

Proteins were extracted from tissue samples and cells using RIPA lysis buffer. The protein concentration was measured using the BCA protein assay kit (Beyotime Biotechnology, Nantong, China). The protein samples were separated by SDS/PAGE, transferred to PVDF membranes (Millipore), and then blocked with 5% non‐fat milk in TBST buffer (tris buffer saline containing 0.1% Tween‐20) for 1 h at room temperature. The membranes were incubated with the indicated primary antibodies overnight at 4 °C, followed by incubation with HRP‐labeled secondary antibodies for 1 h at room temperature. After extensive washing, the bands were visualized using the enhanced chemiluminescence reagent ECL (Millipore).

### 2.8 Quantitative RT‐PCR

The cell samples were homogenized in Trizol reagent (Invitrogen, Oxford, UK). cDNAs were synthesized from 1 μg of extracted total RNA using M‐MLV Reverse Transcriptase kit (Takara, Beijing, China) according to the manufacturer's instructions. Quantitative PCR was performed with SYBR‐Green premix ExTaq (Roche, Shanghai, China) and detected by a real‐time PCR system using the following gene‐specific primer sets: *PELI1* forward, 5′‐CGGCTCAGCAGAGAGGAAAA‐3′, *PELI1* reverse, 5′‐TCACGGTAGGAGTGTGGGAA‐3′; *BclXL* forward, 5′‐ACTCTTCCGGGATGGGGTAA‐3′, *BclXL* reverse, 5′‐AGGTAAGTGGCCATCCAAGC‐3′. The relative gene expressions were assessed in triplicate, normalized to a reference gene *Actb* (encoding β‐actin), and expression levels were determined based on the $2^{‐\Delta \Delta C_{t}}$ method.

### 2.9 *In* *vivo* small animal PET/CT imaging

*In vivo* small animal PET/CT imaging scans and image analysis were performed using a Siemens Inveon Animal‐PET/CT. Mice (*n* = 4) with ESCC were scanned 1 h after injection with the radiolabeled tracer via the tail vein with 100 μCi ^18^F‐FDG. In addition, mice in the ^18^F‐FDG group underwent 4 h of fasting before tracer injection. For the detection of tumor apoptosis *in vivo*, the tumor‐bearing mice were irradiated with a medical linear accelerator (Varian Clinic 23EX; Varian Medical Systems, Palo Alto, CA, USA) using 6 MV photons with an absorption dose rate of 4 Gy·min^−1^ (10 Gy, X‐ray). The tumor‐bearing mice were scanned before or 48 h after irradiation using the radiolabeled tracer ^18^F‐ML‐10. During scanning, the mice were maintained with anesthetization using 2.5% isoflurane/oxygen. During the acquisition of raw images, the three‐dimensional ordered‐subset expectation maximization (OSEM3D)/maximum algorithm was used for image reconstruction. Tracer uptake was determined as the mean and maximum % injected dose pr. gram of tumor (%ID/g) (1 g·cm^−3^). The standard uptake value (SUV) of the tumor was measured by the manually drawn region of the interest method.

### 2.10 Immunohistochemistry

The collected cancer and matched normal tissues were embedded with paraffin, and the paraffin‐embedded tissue sections were then deparaffinized with xylene and rehydrated with diminishing concentrations of alcohol. The endogenous peroxidase activity in the tissues was then blocked with 3% H_2_O_2_ at room temperature for 15 min. Antigen retrieval was performed at a high temperature in sodium citrate (pH 6.0) for 15 min. After slow cooling, the tissue sections were stained with an anti‐PELI1 antibody at 4 °C overnight. After washing with PBS, the sections were incubated with HRP‐labeled goat anti‐mouse IgG for 15 min at room temperature and then stained with the DAB^+^ staining.

Immunohistochemical score was assessed by two independent pathologists without knowledge of patient characteristics as previously described [22]. Briefly, scores were assigned as intensity and percentage of positive staining tumor cell in the whole tissue section. Specifically, the percentage of positive cells was divided into five grades (percentage scores): < 10%, 0; 10–25%, 1; 26–50%, 2; 51–75%, 3; and > 75%, 4. The intensity of the staining was divided into four grades (intensity scores): no staining, 0; light brown, 1; brown, 2; and dark brown, 3. The PELI1 expression level was determined using the following formula: overall scores = percentage score × intensity score. Overall scores of < 6 and ≥ 6 were defined as low and high expression, respectively.

### 2.11 Ubiquitination assay

For the ubiquitination assays, cells were pretreated with MG132 for 2 h and left untreated or IR treated, and then the cells were lysed with cell lysis buffer containing protease inhibitor and *N*‐ethylmaleimide. The cell extracts were boiled for 5 min in the presence of 1% SDS to dissociate the NIK‐interacting proteins and were then diluted with lysis buffer until the concentration of SDS was 0.1% before immunoprecipitation. NIK was then immunoprecipitated from the cell extracts and the immunoprecipitates were immunoblotted with the anti‐Lys48 ubiquitin antibody.

### 2.12 Cell apoptosis analysis

The tumor cells from different genotypes were seeded at the same time and then subdivided into two groups. One group of cells were left nontreated (NT), and another group of cells were irradiated (IR); 24 h after radiotherapy, the NT group and IR group were collected at the same time point for apoptosis analysis. The tumor cell apoptosis was examined using the AnnexinV/PI Apoptosis detection kit (BD Pharmagen, Shanghai, China). Briefly, single‐cell suspensions were stained with Annexin V for 10–15 min at room temperature and protected from light. After washing, the cells were stained with propidium iodide for 5–15 min at room temperature and were then immediately analyzed by flow cytometry. All samples in the same experiments and comparisons were gated under the same parameters.

### 2.13 TUNEL assay

The tumor tissue slides were stained using a TUNEL Apoptosis Detection Kit (A113‐01; Nanjing Vazyme Biotech Co., Ltd, Nanjing, China) for *in situ* detection of apoptosis according to the manufacturer's instructions. Briefly, slides were incubated in the enzyme labeling solution at 37 °C for 1 h in a humidified chamber and then counter‐stained with PBS containing 4,6‐diamidino‐2‐phenylindole (DAPI, 5 μg·mL^−1^) for 5 min. Immunofluorescence results were representative of at least three independent experiments. Images were captured on a confocal microscope (LSM800; ZEISS, Oberkochen, Germany) with fixed settings between samples and quantified using imagej software (Bethesda Softworks LLC, Rockville, MD, USA).

### 2.14 Statistical analysis

Differences between groups were determined using a Student's unpaired *t* test (for two groups), or a two‐way ANOVA with a Bonferroni post‐test when comparing groups with multiple variables. The χ^2^ test was used to assess the immunohistochemical data and clinical information of the patients with the differential expression of PELI1. Survival time was analyzed using the Kaplan–Meier method and compared using the log‐rank test. Univariate and multivariate analyses were based on the Cox proportional hazards regression model. Data are presented as means ± SD. A *P*‐value < 0.05 was considered statistically significant. Statistical details are indicated in the respective figure legends.

## 3 Results

### 3.1 PELI1 predicts the efficacy of adjuvant radiotherapy

**Fig. 1:**
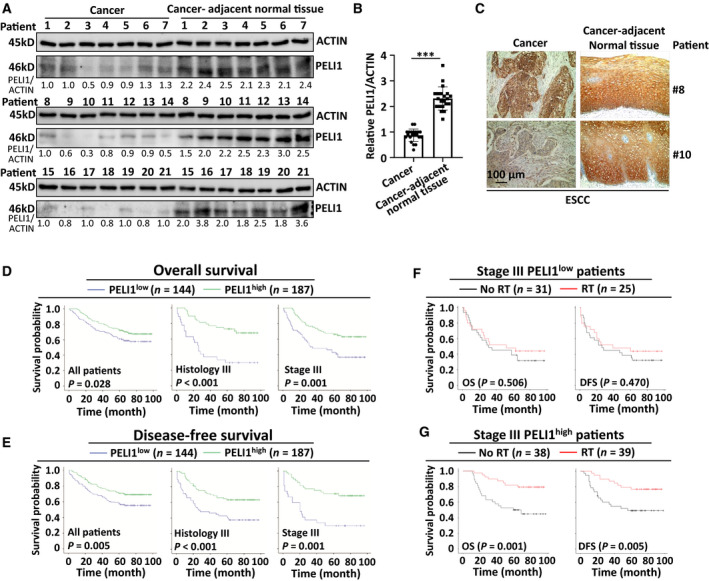
PELI1 is correlated with improved prognosis of human ESCC after radiotherapy. (A, B) Immunoblot analysis and the quantification of pellino E3 ubiquitin protein ligase 1 (PELI1) and ACTIN expression in human ESCC cancer tissues and matched cancer‐adjacent normal tissues (*n* = 21). (C) Representative immunohistochemical staining of PELI1 in human ESCC cancer tissues and matched cancer‐adjacent normal tissues. Scale bar, 100 μm. (D–G) Comparison analysis of the OS and DFS of human ESCC based on PELI1 expression levels (D, E) or with or without radiotherapy among PELI1^low^ or PELI1^high^ patients (F, G) by Kaplan–Meier analysis. The corresponding *n* values and *P* values were indicated in each panel. Data with error bars represent mean ± SEM. *P < 0.05, **P < 0.01, ***P < 0.001, as determined by Student's *t* test (B) or Kaplan–Meier survival analysis (D–G).

To examine the clinical relevance of PELI1 in human ESCC, a total of 331 patients were enrolled, and the patients' characteristics were shown in Table 1. We found that PELI1 expression was dramatically lower in human ESCC and other types of squamous cell carcinomas cancer tissues than their adjacent normal tissues either through immunohistochemical staining of archived paraffin‐embedded tissues or immunoblotting of the collected fresh samples (Fig. 1A–C, Fig. S1). Based on the histological staining, the percentage of PELI1 positive cells was divided into five grades (percentage scores): < 10%, 0; 10–25%, 1; 26–50%, 2; 51–75%, 3; and > 75%, 4. The intensity of the PELI1 staining was divided into four grades (intensity scores): no staining, 0; light brown, 1; brown, 2; and dark brown, 3. The PELI1 expression level was determined using the following formula: overall scores = percentage score × intensity score. Overall scores of < 6 and ≥ 6 were defined as low and high expression, respectively. The rate of PELI1 high expression was 56.5% (187/331) in ESCC cancer tissues, which was significantly lower than the 96.1% in matched cancer–adjacent normal tissues (318/331, *P* < 0.001). High PELI1 expression in patients corresponded with a significantly low proportion of tumors ≥ 4 cm in length compared to those with low expression (*P* < 0.05) (Table 1). These data suggest the decrease of PELI1 expression may contribute to ESCC development.

To further investigate the clinical significance of PELI1 expression in human ESCC, we analyzed the association between the level of PELI1 expression and OS and DFS. As shown in Fig. 1D,E, patients with high PELI1 expression (PELI1^high^) had significantly better OS and DFS than those with low expression (PELI1^low^) (OS: *P* = 0.028; DFS: *P* = 0.005), and these differences were especially prominent in subgroups of histology grade III (OS: *P* < 0.001; DFS: *P* < 0.001) and stage III (OS: *P* = 0.001; DFS: *P* = 0.001). Multivariate models verified that the PELI1 expression level was an independent predictor for OS (HR = 0.58; 95% CI: 0.40–0.83; *P* = 0.003; Table 2) and DFS (HR = 0.52; 95% CI: 0.36–0.76; *P* = 0.001; Table 3).

**Table 2:** Univariate and multivariate analyses of variables associated with overall survival in patients with esophageal squamous cell carcinoma.

Variables	Overall survival			
	Univariate		Multivariate^a^	
	HR (95% CI)	*P* value	HR (95% CI)	*P* value
Age (≥ 65 vs < 65 years)	1.32 (0.92–1.90)	0.129	–	–
Sex (male vs female)	1.45 (0.96–2.19)	0.081	–	–
Tumor location (middle and lower vs upper thoracic)	0.91 (0.48–1.74)	0.775	–	–
Lesion length (≥ 4 vs < 4 cm)^b^	1.35 (0.94–1.93)	0.106	–	–
Histology grade (III vs I/II)	1.62 (1.09–2.42)	0.017	1.71 (1.14–2.57)	0.009
TNM stage (III vs I/II)	2.08 (1.45–2.98)	< 0.001	2.12 (1.47–3.05)	< 0.001
PELI1 expression (high vs low)	0.67 (0.47–0.96)	0.029	0.58 (0.40–0.83)	0.003

^a^ Variables were adopted for their prognostic significance by univariate analysis.

^b^ Median length as a cutoff.

**Table 3:** Univariate and multivariate analyses of variables associated with disease‐free survival in patients with esophageal squamous cell carcinoma.

Variables	Disease‐free survival			
	Univariate		Multivariate^a^	
	HR (95% CI)	*P* value	HR (95% CI)	*P* value
Age (≥ 65 vs < 65 years)	1.37 (0.96–1.96)	0.082	–	–
Sex (male vs female)	1.35 (0.90–2.03)	0.140	–	–
Tumor location (middle and lower vs upper thoracic)	0.93 (0.49–1.79)	0.848	–	–
Lesion length (≥ 4 vs < 4 cm)^b^	1.41 (0.98–2.01)	0.061	–	–
Histology grade (III vs I/II)	1.59 (1.07–2.36)	0.023	1.72 (1.15–2.58)	0.009
TNM stage (III vs I/II)	2.00 (1.40–2.86)	< 0.001	2.06 (1.44–2.96)	< 0.001
PELI1 expression (high vs low)	0.61 (0.42–0.86)	0.006	0.52 (0.36–0.76)	0.001

^a^ Variables were adopted for their prognostic significance by univariate analysis.

^b^ Median length as a cutoff.

We further analyzed the association of the PELI1 expression level with the efficacy of adjuvant radiotherapy in patients with ESCC of stage III (Table 4). As shown in Fig. 1F,G, adjuvant radiotherapy significantly improved OS and DFS of patients with resected stage III ESCC in PELI1^high^ patients (*P* < 0.05) but not in PELI1^low^ patients (*P* > 0.05). The multivariate models showed that adjuvant radiotherapy was an independent predictor for both OS (HR = 0.19; 95% CI 0.07–0.51; *P* = 0.001) and DFS (HR = 0.26; 95% CI 0.11–0.64; *P* = 0.003) in patients with resected stage III ESCC and PELI1^high^ expression (Table 5). These results suggest that PELI1 is a predictor for the efficacy of adjuvant radiotherapy in resected stage III ESCC.

**Table 4:** Patient characteristics according to radiotherapy status in patients with stage III esophageal squamous cell carcinoma.

Patient characteristic	Adjuvant radiotherapy		*P* value
	Untreated (*n* = 69)	Treated (*n* = 64)	
Age (years)			
< 65	39 (56.5)	39 (60.9)	0.605
≥ 65	30 (43.5)	25 (39.1)	
Sex			
Female	14 (20.3)	18 (28.1)	0.291
Male	55 (79.7)	46 (71.9)	
Tumor location			
Upper thoracic	5 (7.2)	9 (14.1)	0.201
Middle and lower thoracic	64 (92.8)	55 (85.9)	
Lesion length (cm)^a^			
< 4	32 (46.4)	36 (56.3)	0.255
≥ 4	37 (53.6)	28 (43.7)	
Histology grade			
I/II	47 (68.1)	48 (75.0)	0.380
III	22 (31.9)	16 (25.0)	
PELI1 expression			
Low	31 (44.9)	25 (39.1)	0.494
High	38 (55.1)	39 (60.9)	

^a^ Median length as a cutoff.

**Table 5:** Multivariate analyses of variables associated with overall survival in patients with stage III esophageal squamous cell carcinoma of high PELI1 expression.

Variables^a^	Disease‐free survival		Overall survival	
	HR (95% CI)	*P* value	HR (95% CI)	*P* value
Age (≥ 65 vs < 65 years)	0.66 (0.28–1.56)	0.346	0.66 (0.28–1.54)	0.334
Sex (male vs female)	1.97 (0.64–6.06)	0.234	2.69 (0.80–9.02)	0.109
Tumor location (middle and lower vs upper thoracic)	0.22 (0.06–0.72)	0.013	0.15 (0.04–0.53)	0.003
Lesion length^b^ (≥ 4 vs < 4 cm)	0.94 (0.44–2.00)	0.869	0.81 (0.38–1.74)	0.589
Histology grade (III vs I/II)	1.10 (0.48–2.53)	0.816	1.13 (0.49–2.61)	0.771
Adjuvant radiotherapy (treated vs untreated)	0.26 (0.11–0.64)	0.003	0.19 (0.07–0.51)	0.001

^a^ Except adjuvant radiotherapy, no variables were found with prognostic significance by univariate analysis and therefore all variables were adopted in multivariate analysis.

^b^ Median length as a cutoff.

### 3.2 *Peli1* deficiency resists IR‐induced esophageal tumor cell apoptosis *in vivo*

**Fig. 2:**
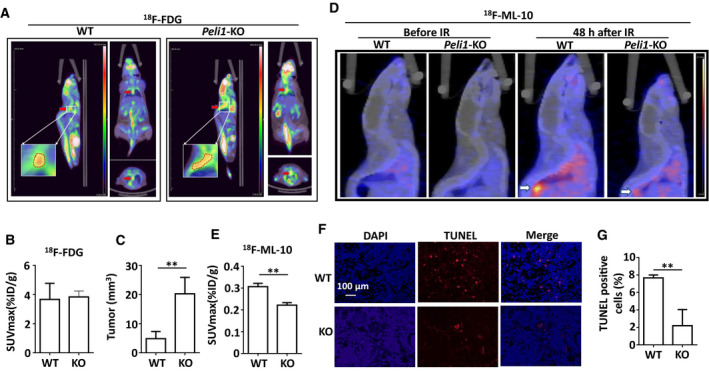
*Peli1* deficiency suppresses IR‐induced apoptosis of ESCC *in vivo*. (A) Small animal positron emission tomography/X‐ray computed tomography (PET/CT) images of different orthogonal sections (sagittal, coronal, transaxial) acquired as static scans 60 min after the injection of 2‐Deoxy‐2‐^18^F‐fluoro‐D‐glucose (^18^F‐FDG) in WT and *Peli1*‐deficient mice induced with ESCC by 4‐nitroquinoline 1‐oxide (4‐NQO) (*n* = 4). The red arrows indicate the tumor. (B, C) Standardized uptake of the ^18^F‐FDG value (SUVmax) and tumor volume of esophageal orthotopic tumors in WT and *Peli1*‐deficient mice that were induced with ESCC by 4‐NQO through PET/CT scanning (*n* = 4). (D) Representative 2‐(5‐^18^F‐fluoropentyl)‐2‐methyl‐malonic acid (^18^F‐ML‐10) PET/CT images of sagittal sections before or 48 h after irradiation (10 Gy/mouse) (*n* = 4). The white arrows indicate the location of tumors. (E) Quantitative analysis of ^18^F‐ML‐10 uptake in tumor tissue expressed as SUVmax 48 h after irradiation (*n* = 4). (F, G) The TUNEL assay showing the apoptosis of 4‐NQO–induced ESCC 48 h after irradiation (10 Gy/mouse; Scale bar, 100 μm; *n* = 4). Each panel is representative of at least two independent experiments. Data with error bars represent mean ± SEM. **P < 0.01, as determined by Student's *t* test (B, C, E, G).

In order to determine the potential function of PELI1 in radiotherapy, we induced a model of esophageal cancer in wild‐type (WT) and *Peli1*‐deficient mice using 4‐nitroquinoline 1‐oxide (4‐NQO) [23]. The results showed that *Peli1* deletion significantly increased the tumorigenesis of 4‐NQO–induced esophageal tumor in mice (Fig. S2). Next, we performed a non‐invasive small animal positron emission tomography/X‐ray computed tomography (PET/CT) analysis after injection with 2′‐deoxy‐2‐^18^F‐fluoro‐d‐glucose (^18^F‐FDG), a clinical drug routinely used to measure the tumor growth based on the glucose metabolism rate [24]. The results revealed that both WT and *Peli1*‐deficient mice showed significant tumor uptake of ^18^F‐FDG as determined by a PET/CT scan, and there was no significant difference in SUVmax between the two groups (3.67 ± 0.90 vs 3.83 ± 0.33, *P* = 0.818) (Fig. 2A,B). However, the results of volume analysis showed that the tumor sizes in the *Peli1*‐deficient mice were significantly larger than those in the WT mice (4.83 ± 2.03 vs 20.23 ± 4.62, *P* = 0.012) (Fig. 2C). These data collectively suggest PELI1 may function as a tumor suppressor gene in esophageal cancer.

To monitor radiotherapy‐induced apoptosis of esophageal tumor cells *in vivo*, we continually performed the small animal PET/CT analysis after injection with 2‐(5‐^18^F‐fluoropentyl)‐2‐methyl‐malonic acid (^18^F‐ML‐10), a small molecular probe used to reveal the degree of cell apoptosis *in vivo* [25]. We observed no significant accumulation of the radiotracer in the esophageal tumors of the WT and *Peli1*‐deficient mice before IR treatment. After undergoing effective radiation therapy, tumor apoptosis was detected by the ^18^F‐ML‐10 PET/CT scan, and the WT mice showed much higher ^18^F‐ML‐10 uptake than did the *Peli1*‐deficient mice (0.31 ± 0.01 vs 0.22 ± 0.01, *P* = 0.002) (Fig. 2D,E). To further confirm that PELI1‐mediated IR‐mediated tumor cell apoptosis in mice, we collected the tumor tissue of IR‐treated tumor‐bearing mice and performed TUNEL immunofluorescence staining. The results showed that TUNEL immunofluorescence in the *Peli1*‐deficient tumor tissue was significantly lower than that in the WT tumor tissue (Fig. 2F,G), indicating that *PELI1* indeed potentiated radiotherapy‐mediated esophageal tumor cell apoptosis *in vivo*.

### 3.3 PELI1 enhances IR‐induced apoptosis of esophageal tumor cells

**Fig. 3:**
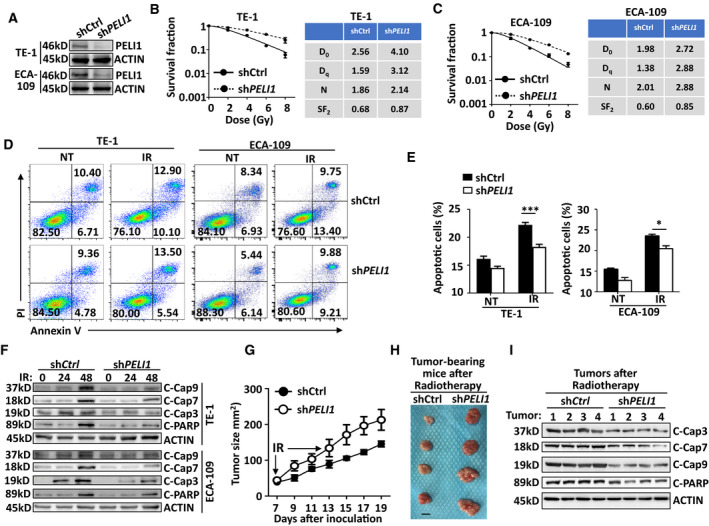
*PELI1* knockdown suppresses IR‐induced cancer cell apoptosis. (A) Immunoblot validation of PELI1 and ACTIN expression in human esophageal squamous cancer cells TE‐1 and ECA‐109 that upon PELI1 knockdown. (B, C) The clonogenic survival assay to examine dose‐dependent survival conditions of control and *PELI1*‐knockdown TE‐1 and ECA‐109 cells upon different doses of IR treatment. Indexes such as D0, Dq, N, and SF2 were calculated and presented in the right panel. (D, E) Flow cytometry analysis of apoptosis frequencies of control and *PELI1*‐knockdown TE‐1 and ECA‐109 cancer cells left non‐treated (NT) or treated with 10 Gy IR. The data are presented as representative plots (D) and a summary bar graph (E) (*n* = 4). (F) Immunoblot analysis of cleaved caspase 3, caspase 9, caspase 7, PARP, and ACTIN (loading controls) in control and *PELI1*‐knockdown TE‐1 and ECA‐109 cancer cells left untreated or treated with 10 Gy IR at the indicated time points. (G–I) Tumor growth curve (G), representative tumor size (H) and *ex vivo* immunoblot of apoptotic signaling in tumors tissues (I) of nude mice that s.c. injected with control or *PELI1*‐knockdown TE‐1 cancer cells and then received 10 Gy IR treatment (IR) at day 7 and day 13 post tumor cell inoculation (*n* = 4 mice/group). Scale bar, 0.5 cm. Each panel is representative of at least three independent experiments. Data with error bars represent mean ± SEM. *P < 0.05, ***P < 0.001, as determined by two‐way ANOVA with a Bonferroni post‐test (E).

**Fig. 4:**
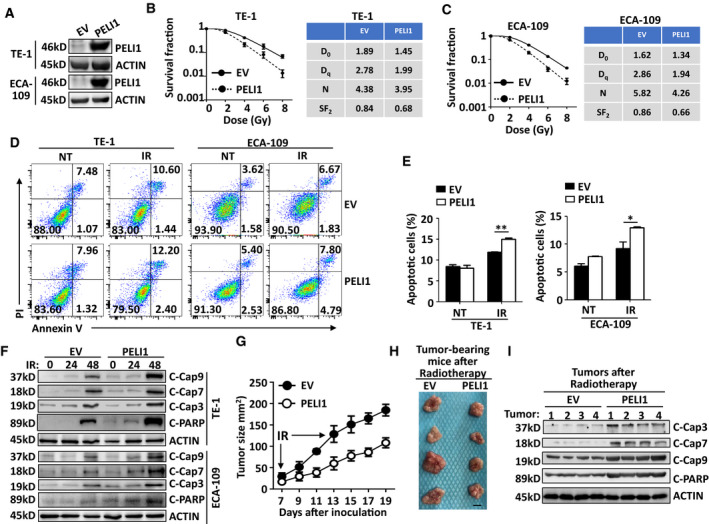
*PELI1* overexpression promotes IR‐induced cancer cell apoptosis. (A) Immunoblot validation of PELI1 and ACTIN expression in human esophageal squamous cancer cells TE‐1 and ECA‐109 that upon PELI1 overexpression. (B, C) The clonogenic survival assay to examine dose‐dependent survival conditions of control and *PELI1*‐overexpression TE‐1 and ECA‐109 cells upon different doses of IR treatment. Indexes such as D0, Dq, N, and SF2 were calculated and presented in the right panel. (D, E) Flow cytometry analysis of the apoptosis frequencies of control and *PELI1*‐overexpression in TE‐1 and ECA‐109 cancer cells left non‐treated (NT) or treated with 10 Gy IR (IR). The data are presented as representative plots (D) and a summary bar graph (E) (*n* = 4). (F) Immunoblot analysis of cleaved caspase 3, caspase 9, caspase 7, PARP, and ACTIN (loading controls) in control and *PELI1*‐overexpression TE‐1 and ECA‐109 cancer cells left untreated or treated with 10 Gy IR at the indicated time points. (G–I) Tumor growth curve (G), representative tumor size (H) and *ex vivo* immunoblot of apoptotic signaling in tumors tissues (I) of nude mice that s.c. injected with control or *PELI1*‐overexpression in TE‐1 cancer cells, and then received 10 Gy IR treatment (IR) at day 7 and day 13 post tumor cell inoculation (*n* = 4 mice/group). Scale bar, 0.5 cm. Each panel is representative of at least three independent experiments. Data with error bars represent mean ± SEM. *P < 0.05, **P < 0.01, as determined by two‐way ANOVA with a Bonferroni post‐test (E).

To confirm the function of PELI1 in modulating IR‐induced apoptosis in ESCC, we examined whether IR treatment could modulate PELI1 expression. The results revealed that IR did not affect the PELI1 expression at both mRNA and protein levels in TE‐1, ECA‐109 and other two types of human squamous cell carcinoma cell lines SCC‐9 (tongue squamous cell line) and SiHa (cervical squamous cancer cell line) (Fig. S3A–D). Next, we performed a clonogenic survival assay to examining the survival conditions of esophageal tumor cells by using different doses of irradiation *in vitro*. The results suggested that *PELI1* knockdown greatly improved the survival condition of both TE‐1 and ECA‐109 cells in response to different doses of irradiation (Fig. 3A–C). Accordingly, although PELI1 did not affect the basal apoptosis levels, IR‐induced apoptosis was also significantly suppressed in both cell lines upon *PELI1* knockdown (Fig. 3D,E), suggesting PELI1 is critical for the regulation of IR‐induced apoptosis whereas it is dispensable for the basal apoptosis rate without irradiation in esophageal tumor cells. In addition, PELI1 knockdown significantly inhibited IR‐induced activation of apoptotic signaling pathways in TE‐1 and ECA‐109 tumor cells, which was reflected by the inhibition of the cleavage of caspase 9, caspase 7, caspase 3, and PARP (Fig. 3F). To confirm PELI1 function *in vivo*, we subcutaneously inoculated the *PELI1*‐knockdown or control cells into nude mice and then treated the tumor‐bearing mice with radiotherapy. The results revealed that the tumor size was bigger, and apoptotic signaling activation was impaired in the *PELI1*‐knockdown TE‐1‐tumor–bearing mice that had undergone two rounds of radiotherapy as compared to the control TE‐1‐tumor–bearing mice (Fig. 3G–I). In contrast, overexpression of PELI1 dramatically impaired the clonogenic ability in response to different doses of irradiation and thus promoted IR‐induced apoptosis of these tumor cells (Fig. 4A–E), enhanced IR‐induced cleavage of caspase 9, caspase 7, caspase 3, and PARP in TE‐1 and ECA‐109 tumor cells both *in vitro* and *in vivo*, and greatly decreased the tumor size after radiotherapy (Fig. 4F–I).

To confirm the pro‐apoptotic function of PELI1, we examined the role of PELI1 in regulating IR‐induced apoptosis in other two types of human squamous cell carcinoma cell lines SCC‐9 and SiHa. In consistent with the data that collected from ESCC cells, PELI1 knockdown also significantly suppressed IR‐induced apoptosis, whereas overexpression of PELI1 dramatically promoted IR‐induced apoptosis in SCC‐9 and SiHa cells (Fig. S3E–L). Collectively, these data establish PELI1 as a tumor intrinsic regulator of IR‐induced apoptosis.

### 3.4 PELI1 inhibits IR‐induced activation of noncanonical NF‐κB through mediating NIK ubiquitination

**Fig. 5:**
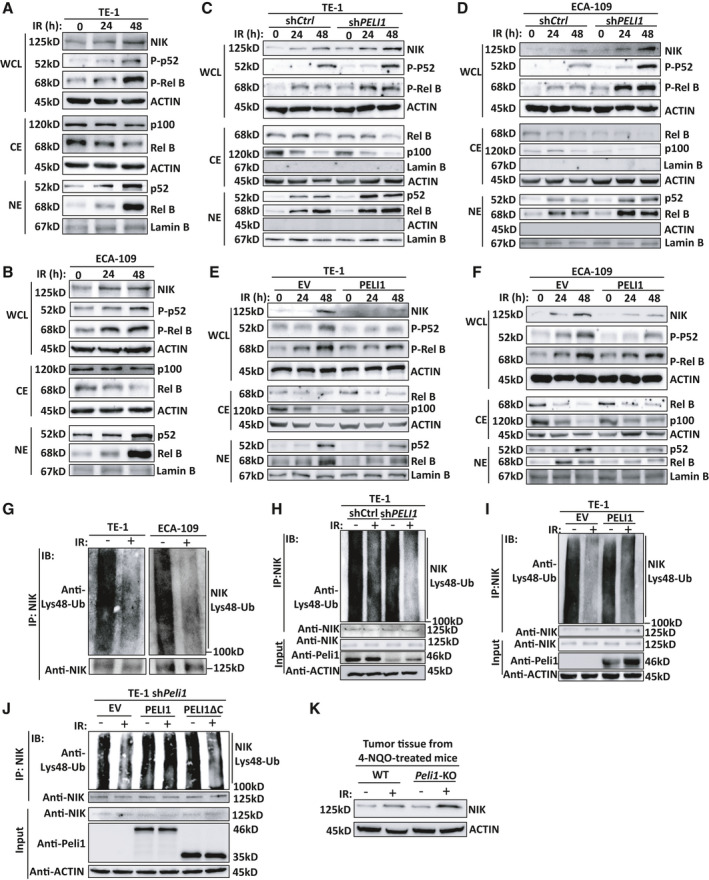
PELI1 negatively regulates IR‐induced noncanonical NF‐κB activation. (A, B) Immunoblot analysis of phosphorylated and total NF‐κB proteins, NIK or ACTIN, and lamin B (loading controls) in whole cell lysates (WCL), cytoplasmic extracts (CE), and nuclear extracts (NE) of TE‐1 (A) and ECA‐109 (B) cells. (C–F) Immunoblot analysis of phosphorylated and total NF‐κB proteins, NIK, ACTIN, and lamin B (loading controls) in cytoplasmic extracts (CE) and nuclear extracts (NE) of *PELI1*‐knockdown or *PELI1*‐overexpression TE‐1 (C, E) and ECA‐109 (D, F) cancer cells left untreated or treated with 10 Gy IR at the indicated time points. (G) Analysis of Lys48 ubiquitination of NIK in TE‐1 and ECA‐109 cancer cells left untreated (−) or treated with 10 Gy IR (+) in the presence of a proteasome inhibitor MG132. IP, immunoprecipitation; IB, immunoblotting. (H, I) Analysis of Lys48 ubiquitination of NIK in *PELI1*‐knockdown (H) or *PELI1*‐overexpression (I) in TE‐1 cancer cells that left untreated (−) or treated with 10 Gy IR (+) in the presence of a proteasome inhibitor MG132. IP, immunoprecipitation; IB, immunoblotting. (J) Analysis of Lys48 ubiquitination of NIK in *PELI1*‐knockdown in TE‐1 cancer cells that reconstituted with empty vector (EV), full‐length PELI1, or C‐terminal deleted PELI1 (PELI1ΔC), and then left untreated (−) or treated with 10 Gy IR (+) in the presence of a proteasome inhibitor MG132. (K) Immunoblot analysis of NIK and ACTIN (loading controls) in tumor tissue collected from 4‐NQO‐treated WT and *Peli1*‐KO mice (*n* = 4 mice/group). Each panel is representative of at least three independent experiments.

To dissect the molecular mechanism controlling PELI1‐mediated apoptosis during radiotherapy, we examined the activation of multiple signaling pathways. Unexpectedly, we observed that neither *PELI1* knockdown nor overexpression affected IR‐induced activation of canonical NF‐κB (including p65, p50 and c‐Rel that highly expressed in B and T lymphocytes) and AKT signaling, two well‐known pathways that mediate tumor cell apoptosis, in the TE‐1 and ECA‐109 cells (Fig. S4A,B). Published studies suggested that PELI1 is a critical negative regulator of B‐cell survival and apoptosis through modulating noncanonical NF‐κB signaling [14]. Therefore, we speculated that PELI1 may also regulate IR‐induced apoptosis through regulating the noncanonical NF‐κB signaling in tumor cells. The results showed that IR could significantly increase the accumulation of NIK protein, a master kinase of the noncanonical NF‐κB signaling pathway, in ESCC TE‐1 and ECA‐109 cells and in SCC‐9 and SiHa squamous carcinoma cells (Fig. 5A,B, Fig. S4C). In addition, IR treatment induced a dramatic phosphorylation and nuclear translocation of p52 and RelB, indicating that IR indeed induced the activation of noncanonical NF‐κB through accumulation of NIK (Fig. 5A,B). As expected, *PELI1* knockdown significantly promoted the accumulation of NIK proteins and thus enhanced the activation of the IR‐induced noncanonical NF‐κB signaling pathway in TE‐1, ECA‐109, SCC‐9 and SiHa squamous carcinoma cells, resulting in enhanced p52 and RelB entry into the nucleus (Fig. 5C,D, Fig. S4D). In contrast, overexpression of *PELI1* impaired the accumulation of NIK proteins and thus inhibited IR‐induced activation of the noncanonical NF‐κB signaling pathway (Fig. 5E,F, Fig. S4E). These data suggest PELI1 has a unique intrinsic function to regulate noncanonical NF‐κB signaling in tumor cells during radiotherapy.

The protein level of NIK in cells is tightly controlled by ubiquitination [5, 6, 7]. In resting cells, NIK is degraded by Lys48‐linked polyubiquitination, thereby maintaining low cellular levels of NIK and shutting down the noncanonical NF‐κB signaling pathway. We found that the Lys48‐linked polyubiquitination of NIK in TE‐1 and ECA‐109 tumor cells after IR treatment was significantly reduced (Fig. 5G), which also helped to explain why irradiation induced NIK protein accumulation and activation of the noncanonical NF‐κB signaling pathway. In addition, IR‐induced NIK accumulation and difference between control and *PELI1*‐knockdown cells were abolished when pretreated with protein synthesis inhibitor cycloheximide (Fig. S4F), suggesting PELI1‐mediated NIK protein difference might be controlled by ubiquitination‐induced degradation.

Previous studies have shown that PELI1 is a direct E3 ubiquitin ligase of NIK and mediates the ubiquitination and degradation of NIK in B cells [14]. Since IR treatment inhibited the ubiquitination of NIK and activated the noncanonical NF‐κB signaling pathway, we next investigated the effect of the regulation of PELI1 on NIK during radiotherapy. The results indicated that knockdown of *PELI1* in TE‐1, SCC‐9 or SiHa tumor cells further promoted the reduction of IR‐induced Lys48‐linked polyubiquitination of NIK (Fig. 5H, Fig. S4G), whereas overexpression of *PELI1* in tumor cells significantly inhibited the IR‐induced reduction of Lys48‐linked polyubiquitination of NIK (Fig. 5I, Fig. S4H). In addition, reconstitution of full‐length PELI1 in *PELI1*‐knockdown TE‐1 cells dramatically recovered NIK ubiquitination after irradiation, whereas reconstitution of PELI1 RING domain mutant (PELI1ΔC, lack of E3 ligase activity) has no recovery effect on NIK ubiquitination as empty vector (EV) did after irradiation (Fig. 5J). Moreover, IR‐induced NIK accumulation was also dramatically promoted in tumor tissue from 4‐NQO–treated *Peli1*‐KO mice as compared with that in WT mice (Fig. 5K). These data collectively suggest that PELI1 negatively regulates IR‐induced activation of noncanonical NF‐κB through mediating NIK ubiquitination and degradation *in vitro* and *in vivo*.

### 3.5 PELI1 mediates IR‐induced apoptosis through inhibition of Bcl‐XL

**Fig. 6:**
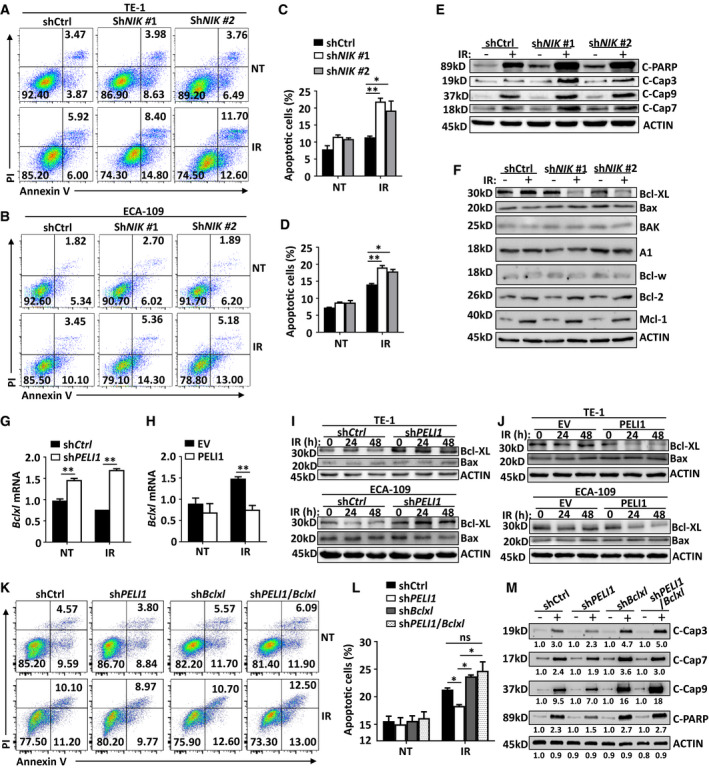
PELI1 mediates IR‐induced apoptosis through inhibition of Bcl‐XL. (A‐D) Flow cytometry analysis of the apoptosis frequencies of control and *NIK*‐knockdown TE‐1 and ECA‐109 cancer cells left non‐treated (NT) or treated with 10 Gy IR (IR). The data are presented as representative plots (A, B) and a summary bar graph (C, D) (*n* = 4). (E, F) Immunoblot analysis of cleaved PARP, caspase 3, caspase 9, caspase 7, Bcl‐XL, Bax, BAK, A1, Bcl‐w, Bcl‐2, Mcl‐1 and ACTIN (loading controls) in control and *NIK*‐knockdown TE‐1 cancer cells left non‐treated or treated with 10 Gy IR. (G, H) Quantitative PCR analysis of *Bclxl* mRNA expression in *PELI1*‐knockdown or *PELI1*‐overexpression in TE‐1 cancer cells left non‐treated (NT) or treated with 10 Gy (*n* = 4). (I, J) Immunoblot analysis of Bcl‐XL, Bax, and ACTIN (loading controls) in *PELI1*‐knockdown (I) or *PELI1*‐overexpression (J) in TE‐1 and ECA‐109 cancer cells left untreated or treated with 10 Gy at the indicated time points. (K, L) Flow cytometry analysis of the apoptosis frequencies of control, *PELI1*‐knockdown, *Bclxl*‐knockdown, or *PELI1*/*Bclxl*‐knockdown in TE‐1 cancer cells that were non‐treated (NT) or treated with 10 Gy IR (IR). The data are presented as representative plots (K) and a summary bar graph (L) (*n* = 4). (M) Immunoblot analysis of cleaved PARP, caspase 3, caspase 9, caspase 7, and ACTIN (loading controls) in control, *PELI1*‐knockdown, *Bclxl*‐knockdown, or *PELI1*/*Bclxl*‐knockdown TE‐1 cancer cells left untreated or treated with 10 Gy IR. Each panel is representative of at least three independent experiments. Data with error bars represent mean ± SEM. *P < 0.05, **P < 0.01, as determined by two‐way ANOVA with a Bonferroni post‐test (C, D, G, H, L).

To confirm the apoptosis‐resistant effect of IR‐induced noncanonical NF‐κB signaling, we reduced NIK expression and examined IR‐induced tumor cell apoptosis. As expected, NIK knockdown significantly increased the frequency of IR‐induced apoptosis in both TE‐1 and ECA‐109 cancer cells (Fig. 6A–D). Accordingly, knockdown of NIK also dramatically promoted the activation of the IR‐induced apoptosis signaling pathway, manifested by significantly increased cleavage of caspase 9, caspase 7, caspase 3, and PARP (Fig. 6E). These results suggest IR‐induced noncanonical NF‐κB signaling indeed functions as a negative regulator of IR‐mediated apoptosis during radiotherapy.

Recently published work has shown that noncanonical NF‐κB signaling modulates the transcription of apoptosis‐related genes, including *Bclx1* [14], of which the anti‐apoptotic protein Bcl‐XL can suppress the occurrence of apoptosis by inhibiting the cleavage of caspase 9 [26]. We thusly hypothesized that noncanonical NF‐κB‐mediated apoptosis resistance during radiotherapy was due to the induction of Bcl‐XL. Indeed, we found that the noncanonical NF‐κB signaling specifically mediated the expression of *Bclx1*, as demonstrated by dramatically decreased Bcl‐XL protein levels without affecting other apoptosis‐related proteins in the NIK‐knockdown tumor cells after IR treatment (Fig. 6F), suggesting NIK‐mediated noncanonical NF‐κB is dispensable for irradiation‐induced Mcl‐1 and Bcl‐2 protein expression. In concert with the pattern of PELI1‐modulated noncanonical NF‐κB signaling, knockdown of PELI1, which enhanced IR‐induced noncanonical NF‐κB signaling, significantly promoted the transcription of the anti‐apoptotic gene *Bclx1* in IR‐treated TE‐1 tumor cells (Fig. 6G). However, the overexpression of PELI1, which impaired IR‐induced noncanonical NF‐κB signaling, dramatically inhibited the transcription of this gene in tumor cells (Fig. 6H). Correspondingly, *PELI1* knockdown specifically promoted the expression of the Bcl‐XL protein in IR‐treated TE‐1, ECA‐109, SCC‐9 and SiHa squamous carcinoma cells (Fig. 6I, Fig. S5A), while overexpression of PELI1 also reduced the Bcl‐XL protein levels in these tumor cells (Fig. 6J, Fig. S5B). In addition, PELI1 knockdown or overexpression does not affect the activation of ATM and p53 before or after IR treatment (Fig. S5C,D).

To confirm the Bcl‐XL function in PELI1‐mediated apoptosis during radiotherapy, we reduced *Bclxl* expression in control and PELI1‐knockdown tumor cells. Interestingly, knockdown of *Bclxl* significantly enhanced IR‐induced tumor cell apoptosis and abolished the apoptosis‐resistant effect in PELI1‐knockdown cells (Fig. 6K,L). Accordingly, knockdown of *Bclxl* promoted the activation of the IR‐induced apoptosis signaling pathway in PELI1‐knockdown cells, which was demonstrated by increased cleavage of caspase 9, caspase 7, caspase 3, and PARP in *PELI1*/*Bclxl*‐knockdown cells (Fig. 6M, Fig. S5E). These results collectively suggest PELI1 negatively regulates noncanonical NF‐κB‐induced Bcl‐XL expression, which further modulate IR‐induced cancer cell apoptosis.

### 3.6 Noncanonical NF‐κB is required for IR‐induced apoptosis resistance in PELI1‐knockdown cells

**Fig. 7:**
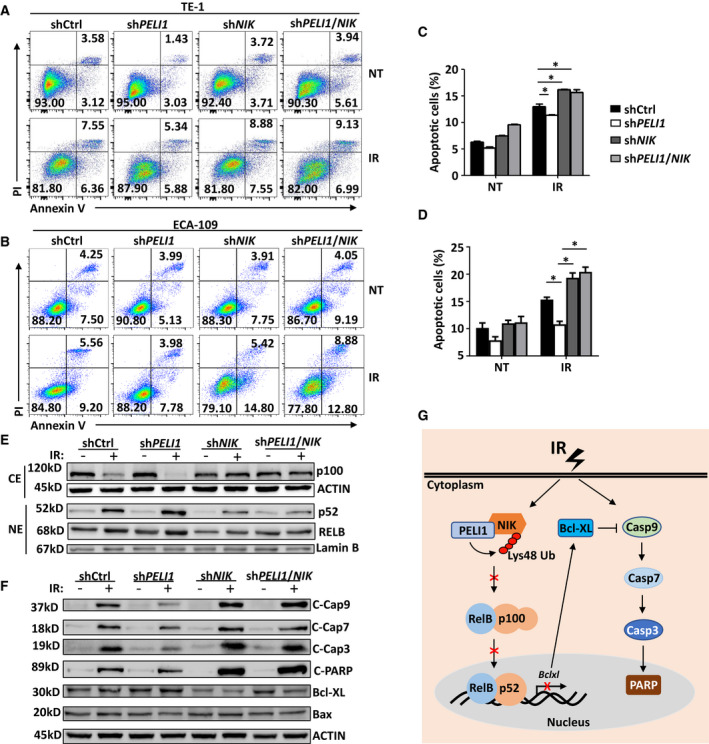
Noncanonical NF‐KB is required for the apoptosis resistance in *PELI1*‐knockdown cells. (A–D) Flow cytometry analysis of the apoptosis frequencies of control, *PELI1*‐knockdown, *NIK*‐knockdown or *PELI1*/*NIK* double knockdown TE‐1 and ECA‐109 cancer cells left non‐treated (NT) or treated with 10 Gy IR (IR). The data are presented as representative plots (A, B) and a summary bar graph (C, D) (*n* = 4). (E, F) Immunoblot analysis of NF‐κB, ACTIN, and lamin B (loading controls) in cytoplasmic extracts (CE) and nuclear extracts (NE) (E), and of the of cleaved PARP, caspase 3, caspase 9, caspase 7, Bcl‐XL, Bax, and ACTIN (loading controls) in whole cell lysates (F) of control, *PELI1*‐knockdown, *NIK*‐knockdown or *PELI1*/*NIK* double knockdown in TE‐1 cancer cells left untreated (−) or treated with 10 Gy IR (+). (G) Proposed model illustrating the mechanism of PELI1 regulating the sensitivity of radiotherapy through modulating noncanonical NF‐κB. Each panel is representative of at least three independent experiments. Data with error bars represent mean ± SEM. *P < 0.05 as determined by two‐way ANOVA with a Bonferroni post‐test (C, D).

Since IR‐induced noncanonical NF‐κB inhibits tumor cell apoptosis and PELI1 negatively regulates IR‐induced activation of this signaling pathway, we speculated that PELI1 may affect IR‐induced apoptosis of tumor cells through modulation of noncanonical NF‐κB signaling. The results revealed that knockdown of NIK, the master protein kinase of the noncanonical NF‐κB signaling pathway, significantly restored the decreased IR‐induced apoptosis in *PELI1*‐knockdown TE‐1 and ECA‐109 tumor cells (Fig. 7A–D). Correspondingly, knockdown of NIK significantly inhibited IR‐induced activation of the non‐canonical NF‐KB signaling pathway in *PELI1*‐knockdown tumor cells, manifested by the inhibition of RelB and p52 nuclear translocation upon IR treatment (Fig. 7E). In addition, knockdown of NIK dramatically promoted IR‐induced cleavage of caspase 9, caspase 7, caspase 3, and PARP in *PELI1*‐knockdown tumor cells (Fig. 7F), which was consistent with the apoptosis flow cytometry assay results. These data collectively suggest noncanonical NF‐κB is required for the resistance of IR‐induced apoptosis in *PELI1*‐knockdown cells.

Based on the above findings, we propose a model to demonstrate the molecular mechanism by which PELI1 modulates radiotherapy‐induced esophageal tumor cell apoptosis through regulating the noncanonical NF‐κB signaling pathway (Fig. 7G). During radiotherapy, IR induces the accumulation of NIK in tumor cells to activate the noncanonical NF‐κB signaling pathway. This activation mediates the expression of the anti‐apoptotic gene *Bclxl*, which suppresses IR‐induced caspase 9 cleavage and maturation, thereby further inhibiting downstream caspase 7, caspase 3, and PARP cleavage and tumor cell apoptosis. However, PELI1 mediates the Lys48‐linked polyubiquitination and degradation of NIK and thus inhibits IR‐induced activation of noncanonical NF‐κB signaling. Therefore, PELI1 deletion enhances IR‐induced activation of the noncanonical NF‐κB signaling pathway to promote the expression of Bcl‐XL, which further diminishes the IR‐induced apoptosis signaling pathway and impairs radiosensitivity.

## 4 Discussion

Recent studies have shown that the noncanonical NF‐κB signaling pathway plays an important regulatory role in immune cells [5, 6, 7], and its intrinsic function in tumor cells has gradually been revealed [8, 9, 10, 11, 12]. For example, the presence of the p100 C‐terminal deleted mutation was found in various tumors, such as multiple myelomas [27], non‐Hodgkin's lymphomas [28], and cutaneous T‐cell lymphomas [29]. The C‐terminus deletion of p100 results in the constitutive processing of p100 to p52, which in turn activates the target genes controlled by noncanonical NF‐κB [5, 6, 7], and therefore promotes tumor cell proliferation and survival. In addition, other mutations affecting upstream kinases and regulators (TRAF3, CD40, BAFFR, and NIK) of this pathway, which promote NIK stability and subsequent activation of noncanonical NF‐κB, have been described in different cancer types [9, 10, 11, 12], including splenic marginal zone lymphomas, Hodgkin's lymphomas, and lung cancer. Here, we have for the first time found that IR activates the noncanonical NF‐κB signaling pathway, which in turn resists IR‐induced esophageal tumor cell apoptosis during radiotherapy. More interestingly, we identified that PELI1, a NIK E3 ubiquitin ligase, mediates the Lys48‐linked polyubiquitination and degradation of NIK during radiotherapy and thus negatively regulates the activation of the IR‐induced noncanonical NF‐κB signaling pathways, thereby promoting the radiotherapy‐induced apoptosis of esophageal tumor cells.

The biological function of the E3 ligase, PELI1, has been well‐characterized in various immune cells, including lymphoid‐derived T and B cells, as well as myeloid‐derived macrophages and microglia; PELI1 regulates specific inflammatory signaling pathways by mediating the Lys48 or Lys63‐linked polyubiquitination of specific substrates, thereby ultimately modulating the proliferation, activation, and inflammatory responses of these immune cells [14, 15, 16, 17, 18, 19]. Recently, the functional study of PELI1 in tumors has shown that the expression of PELI1 in lung adenocarcinoma is significantly higher than that of matched cancer‐adjacent normal tissues, and this contributes to lung tumorigenesis and promotes epithelial–mesenchymal transition by mediating the Lys63‐linked polyubiquitination of Snail and Slug [20]. Therefore, PELI1 acts as an oncoprotein in lung adenocarcinoma to regulate tumor development. However, our present study showed that the expression of PELI1 in ESCC was significantly lower than that of matched normal tissues, and PELI1 functioned as a tumor suppressor to regulate the radiosensitivity in squamous carcinomas cells. In addition, PELI1 has been reported to inhibit the radiosensitivity in osteosarcoma U2OS cells [30]. This functional contradiction of PELI1 may be caused by the essential difference between adenocarcinoma, osteosarcoma and squamous cell carcinomas, which also suggests that PELI1 may functions in a context‐dependent manner as a tumor suppressor in squamous cell carcinomas, whereas it functions as a oncoprotein in adenocarcinoma.

IR‐induced tumor cell apoptosis is the most important mechanism for radiotherapy tumor treatment. Recent evidence has suggested that PELI1 plays an important role in regulating cell survival. PELI1 can specifically target kinase‐active RIPK3 for its Lys48‐linked polyubiquitination and degradation, and thus it effectively inhibits TNF‐induced necroptosis [31]. However, there is also evidence indicating that PELI1 promotes cell death in mouse embryonic fibroblasts by mediating the Lys63‐linked polyubiquitination of RIPK1, which increases the interaction of RIPK1 and RIPK3 and leads to necrosis [32]. Our present study demonstrates that PELI1 exhibits a pro‐apoptotic function through inhibiting IR‐induced activation of noncanonical NF‐κB during radiotherapy, which is similar to its necrosis‐promoting function.

There is no validated prognostic or predictive biomarker for ESCC patients who receive adjuvant radiotherapy, and current treatment selection for these patients is based mainly on postoperative pathological staging. Our study suggests a prognostic and predictive role for PELI1 in radiotherapy. Although prospective validation is necessary, our findings have the potential to improve the treatment selection for adjuvant radiotherapy and may spare a large number of ESCC patients from unnecessary therapy.

## 5 Conclusion

In conclusion, our study identified that IR‐activated noncanonical NF‐κB signaling in tumor cells is critical for tumor resistance to apoptosis during radiotherapy. PELI1 can impair IR‐induced activation of noncanonical NF‐κB by mediating NIK ubiquitination and degradation, thereby promoting the sensitivity of tumors to radiotherapy. In view of the pivotal role of noncanonical NF‐κB signaling and its regulator PELI1 in the regulation of radiosensitivity, targeted interventions of the noncanonical NF‐κB signaling pathway or related regulator PELI1 may have clinical potential for the enhanced sensitivity of radiotherapy.

## Conflict of interest

The authors declare no conflict of interest.

## Author contributions

DD designed and performed the experiments, prepared the figures, and wrote the manuscript; HZ, LY, FY, XY, TY, XZ, WL, and DW contributed to part of the experiments; XH, JF, and DC supervised the work and wrote the manuscript.

### Peer review

The peer review history for this article is available at https://publons.com/publon/10.1002/1878‐0261.13134.

## Supporting information


**Fig. S1.** Immunohistochemical staining of PELI1 in multiple types of human squamous cell carcinomas and matched cancer‐adjacent normal tissues.
**Fig. S2.** Peli1 deficiency promoted 4‐NQO‐induced esophageal squamous tumor growth.
**Fig. S3.** PELI1 potentiates IR‐induced cancer cell apoptosis in multiple human squamous carcinomas cell lines.
**Fig. S4.** PELI1 negatively regulates IR‐induced noncanonical NF‐κB activation and mediates IR‐induced NIK ubiquitination in multiple human squamous carcinomas cell lines.
**Fig. S5.** PELI1 specifically inhibits Bcl‐XL expression in multiple human squamous carcinomas cell lines.

## Data Availability

All data generated or analyzed during this study are included either in this article or in the supporting Figures and Figure Legends files.
