# Supplementary material for: PELI1 promotes radiotherapy sensitivity by inhibiting noncanonical NF‐κB in esophageal squamous cancer
Source: Mol Oncol. 2021 Nov 14;16(6):1384–401. doi: 10.1002/1878-0261.13134 (PMC8936515; doi:10.1002/1878-0261.13134)
Supplement: Supplementary file 1 — Fig. S1. Immunohistochemical staining of PELI1 in multiple types of human squamous cell carcinomas and matched cancer‐adjacent normal tissues. Fig. S2. Peli1 deficiency promoted 4‐NQO‐induced esophageal squamous tumor growth. Fig. S3. PELI1 potentiates IR‐induced cancer cell apoptosis in multiple human squamous carcinomas cell lines. Fig. S4. PELI1 negatively regulates IR‐induced noncanonical NF‐κB activation and mediates IR‐induced NIK ubiquitination in multiple human squamous carcinomas cell lines. Fig. S5. PELI1 specifically inhibits Bcl‐XL expression in multiple human squamous carcinomas cell lines. [file MOL2-16-1384-s001.docx]

**Supporting information**

**PELI1 promotes radiotherapy sensitivity by inhibiting noncanonical NF-κB in esophageal squamous cancer**

by Dai et al.


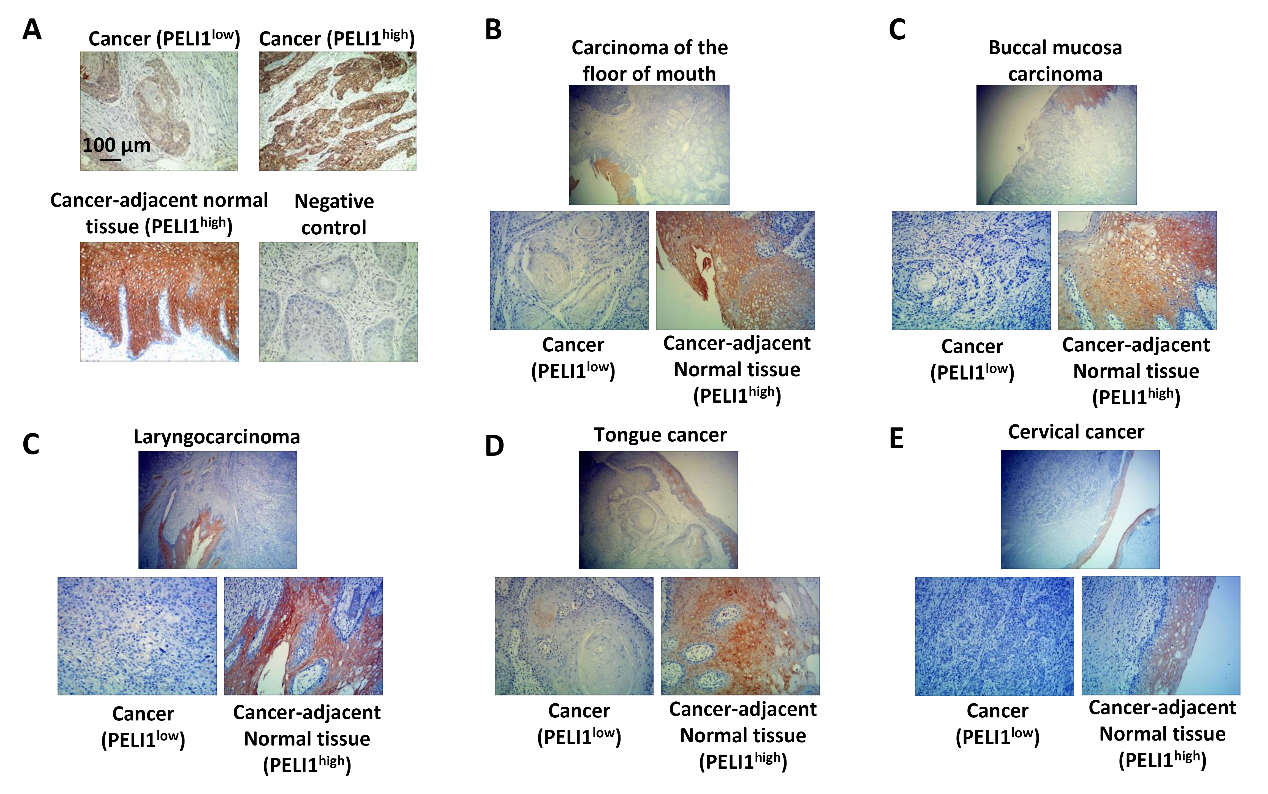


**Figure S1. Immunohistochemical staining of PELI1 in multiple types of human squamous cell carcinomas and matched cancer-adjacent normal tissues.**

**Figure S2. Peli1 deficiency promoted** **4-NQO-induced esophageal squamous tumor growth.**

(**A**) The scheme of 4-NQO-induced esophageal squamous cancer in WT and *Peli1*-deficient mice. (**B**) The images of 4-NQO-induced esophageal squamous tumor in WT and *Peli1*-deficient (KO) mice. (C, **D**) H & E staining of 4-NQO-induced esophageal lesions representative of different stages of carcinogenesis in WT and *Peli1*-deficient mice at different time points. (i) normal epithelium, (ii)mild dysplasia, (iii) moderate-to-severe dysplasia and (iv) esophageal squamous cell cancer. (**E**) The tumorigenesis rates of 4-NQO-induced esophageal squamous tumor in WT and *Peli1*-deficient (KO) mice. The statistical significance was determined by χ^2^ test.


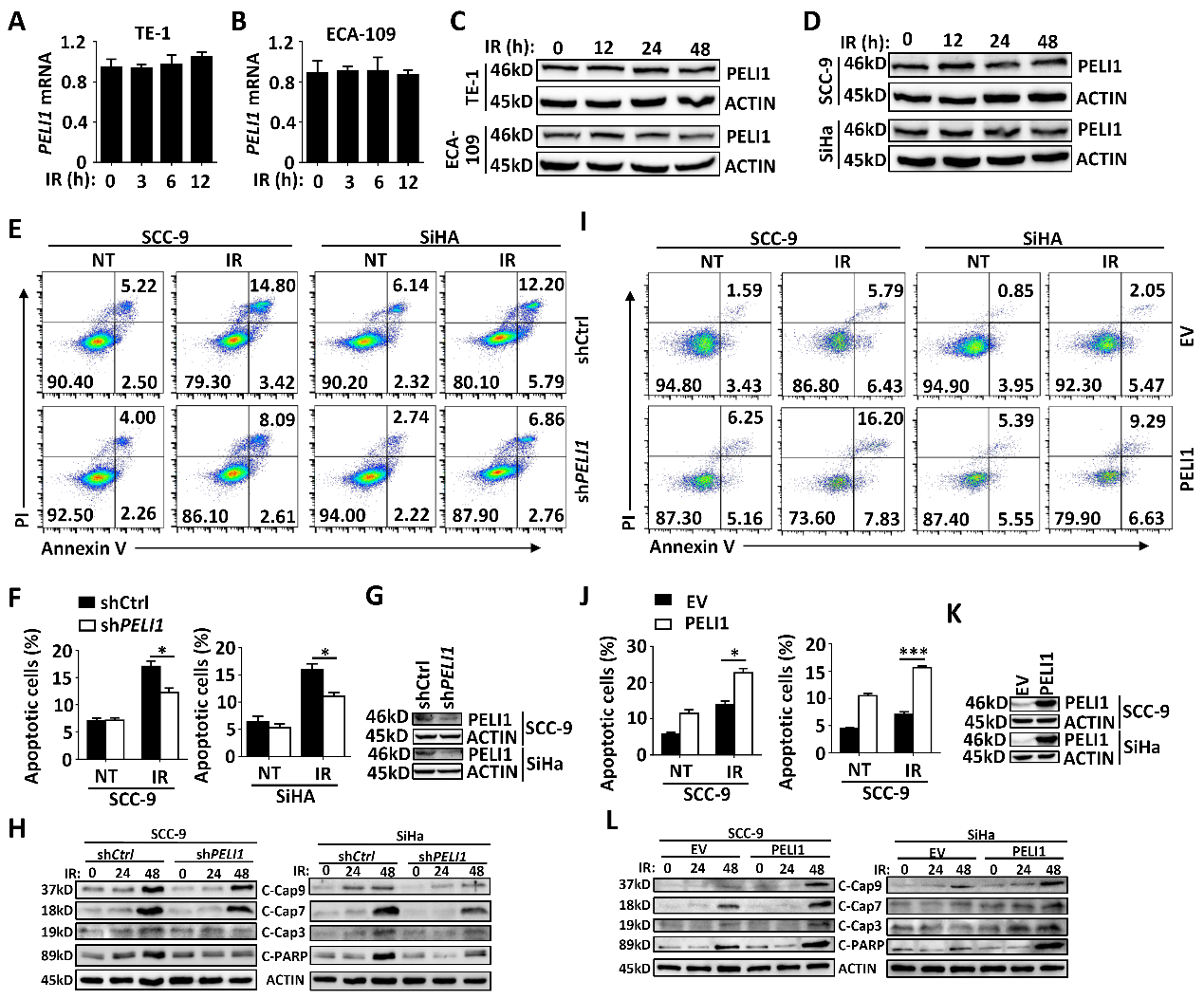


**Figure S3. PELI1 potentiates IR-induced cancer cell apoptosis in multiple human squamous carcinomas cell lines**. (**A, B**) QPCR analysis of *PELI1* mRNA expression in TE-1 and ECA-109 cells that treated with 10 Gy IR with the indicated time points. (**C, D**) Immunoblot of PELI1 and ACTIN expression in TE-1, ECA-109, SCC-9 and SiHa human squamous carcinomas cell lines that treated with 10 Gy IR with the indicated time points. (**E, F**) Flow cytometry analysis of apoptosis frequencies of control and *PELI1*-knockdown SCC-9 and SiHa cancer cells left non-treated (NT) or treated with 10 Gy IR (IR). The data are presented as representative plots (**E**) and a summary bar graph (**F**). (**G**) Immunoblot validation of PELI1 and ACTIN expression in human esophageal squamous cancer cells SCC-9 and SiHa cells that upon PELI1 knockdown. (**H**) Immunoblot analysis of cleaved caspase 3, caspase 9, caspase 7, PARP, and ACTIN (loading controls) in control and *PELI1*-knockdown SCC-9 and SiHa cancer cells left untreated or treated with10 Gy IR at the indicated time points. (**I, J**) Flow cytometry analysis of the apoptosis frequencies of control and *PELI1*-overexpression in SCC-9 and SiHa cancer cells left non-treated (NT) or treated with 10 Gy IR (IR). The data are presented as representative plots (**I**) and a summary bar graph (**J**). (**K**) Immunoblot validation of PELI1 and ACTIN expression in human esophageal squamous cancer cells SCC-9 and SiHa cells that upon PELI1 overexpression. (**L**) Immunoblot analysis of cleaved caspase 3, caspase 9, caspase 7, PARP, and ACTIN (loading controls) in control and *PELI1*-overexpression SCC-9 and SiHa cancer cells left untreated or treated with 10 Gy IR at the indicated time points. Each panel is representative of at least three independent experiments. Data with error bars represent mean ± SEM. ∗P < 0.05, ∗∗∗P < 0.001, as determined by two-way ANOVA with a Bonferroni post test (**F, J**).


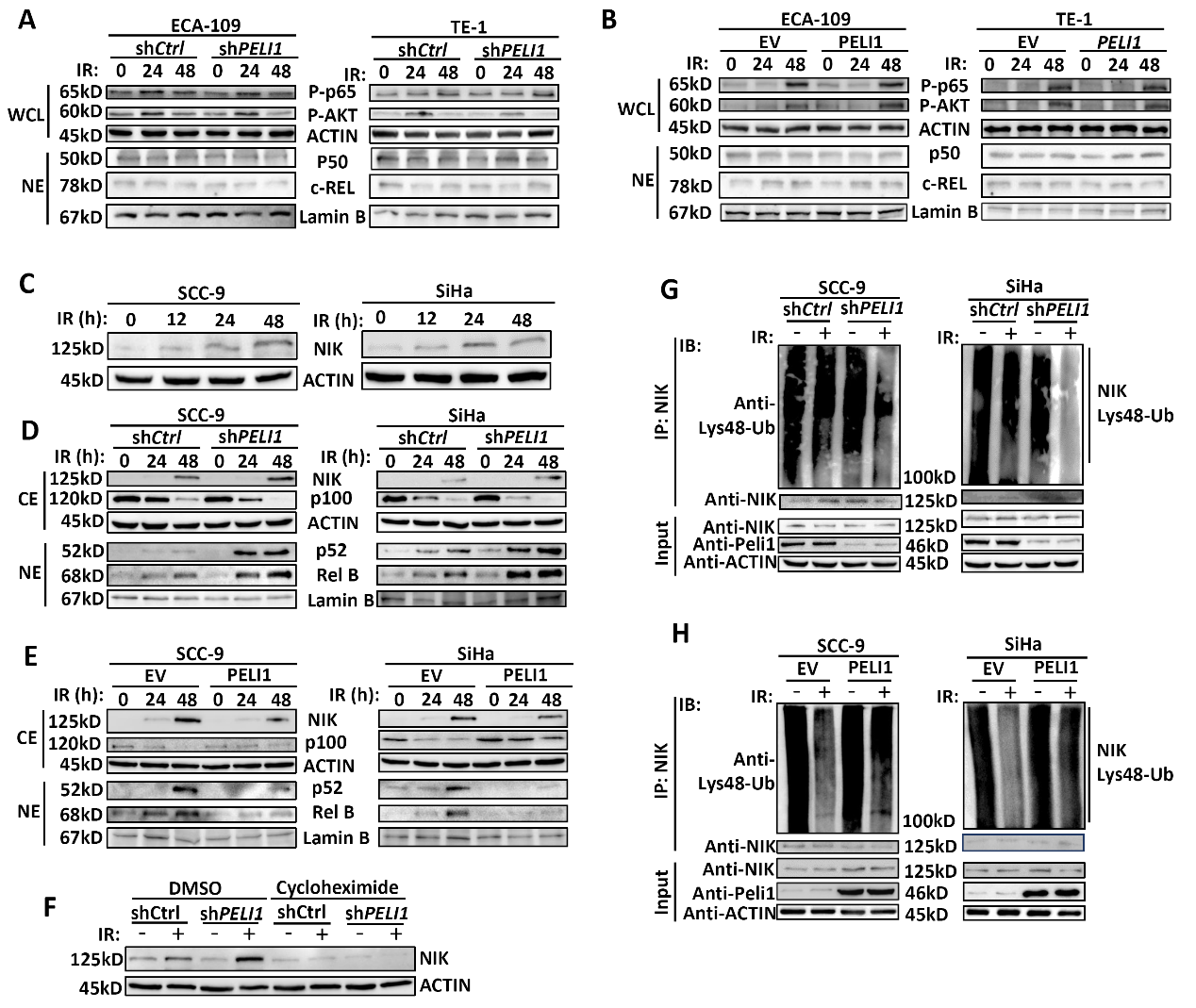


**Figure S4. PELI1 negatively regulates IR-induced noncanonical NF-κB activation and mediates IR-induced NIK ubiquitination in multiple human squamous carcinomas cell lines.** (**A, B**) Immunoblot analysis of phosphorylated (P) p65 and AKT, p50, c-Rel, and ACTIN or Lamin B (loading control) in control and *PELI1*-knockdown ECA-109 and TE-1 cancer cells (**A**) or in control and PELI1-overexpression ECA-109 and TE-1cancer cells (**B**) that left untreated or IR treatment for 10 Gy at the indicated time points. (**C**) Immunoblot analysis of NIK and ACTIN (loading controls) in whole cell lysates of SCC-9 and SiHa cells. (**D, E**) Immunoblot analysis of NF-κB proteins, ACTIN, and lamin B (loading controls) in cytoplasmic extracts (CE) and nuclear extracts (NE) of *PELI1*-knockdown or *PELI1*-overexpression SCC-9 and SiHa cancer cells left untreated or treated with 10 Gy IR at the indicated time points. (**F**) Immunoblot analysis of NIK and ACTIN (loading controls) in whole cell lysates of TE-1 cells that pre-treated with DMSO or cycloheximide for 2 h, and then left untreated or treated with 10 Gy IR for 48 h. (**G, H**) Analysis of Lys48 ubiquitination of NIK in *PELI1*-knockdown (**G**) or *PELI1*-overexpression (**H**) SCC-9 and SiHa cancer cells that left untreated (-) or treated with 10 Gy IR (+) in the presence of a proteasome inhibitor MG132. IP, immunoprecipitation; IB, immunoblotting. Each panel is representative of at least three independent experiments.

**Figure S5. PELI1 specifically inhibits Bcl-XL expression in multiple human squamous carcinomas cell lines.** (**A, B**) Immunoblot analysis of Bcl-XL and ACTIN (loading controls) in *PELI1*-knockdown (**A**) or *PELI1*-overexpression (**B**) in SCC-9 and SiHa cancer cells left untreated or treated with 10 Gy at the indicated time points. (**C, D**) Immunoblot analysis of phosphorylated (P) ATM and p53, and ACTIN (loading control) in control and *PELI1*-knockdown TE-1 and ECA-109 cancer cells (**C**) or in control and *PELI1*-overexpression ECA-109 and TE-1cancer cells (**D**) that left untreated or IR treatment for 10 Gy at the indicated time points. Each panel is representative of at least three independent experiments. (**E**) Immunoblot analysis (upper) and quantitative analysis (lower) of cleaved PARP, caspase 3, caspase 9, caspase 7, and ACTIN (loading controls) in control, *PELI1*-knockdown, *Bclxl*-knockdown, or *PELI1*/*Bclxl*-knockdown TE-1 cancer cells left untreated or treated with 10 Gy IR.
